# Teaching-learning: An integral component of sound patient care

**DOI:** 10.4103/0019-5413.41846

**Published:** 2008

**Authors:** Anil K Jain

**Affiliations:** Professor of Orthopedics, University College of Medical Sciences, University of Delhi, Delhi-110 095, India. and Editor Indian Journal of Orthopaedics. E-mail: dranilkjain@gmail.com

The present generation of clinicians is able to draw better conclusions on the past practices and use them to alleviate pain, suffering and prolong the life expectancy of human beings. The sound training of the future generation of health personnel and learning from the past experiences is most important for continued evolution of medical science and dissemination of the knowledge far and wide.

The present issue of Indian Journal of Orthopedics has two writings on orthopedic education. The Orthopedic education is far more organized in United States and Europe and now some of the finer issues such as mentorship,^1^ residency program, need for research training and research rotation,^2^ optimizing the learning, providing an environment for progressive learning,^3^ how to teach technical skills to surgical residents,^4^ use of simulation techniques in training,^5^ effect of work hour restriction on training^6^ and relationship of industry and education and professionalism^7^ are in discussion. The article by Sarmiento on “the education of orthopedic resident” gives a US perspective reflecting the state of education in the developed nations while we in India are still working on the basics of the core curriculum, quality and uniform delivery of education. We are in an advantageous position to have seen the natural history of orthopedic education in the west, hence we can structure better delivery of education by learning a few lessons from the experience in the US and Europe and making concerted efforts to organize orthopedic education best suited for our patient population in their social milieu. The article by Kumar and Tuli on “postgraduate education-Indian perspective” clearly discusses the lacunae, proposes solutions to correct the deficiencies. Summarily, the orthopedics' specific core competencies need to be defined to train postgraduate for “must know items”. The delivery of training should be improved by constant evaluation of trainee, trainer and frequent reappraisal of the education system. Orthopedics has expanded many-fold in the last 40 years in view of recent advances in imaging, metallurgy, implant technology, diagnostic facilities. Various subspecialties have emerged. The time has come to review the duration of the course to accommodate expanded core curriculum. Post-qualification training as senior registrar should be made available to all orthopedic trainees to develop competence where the trainee learns “when not to operate” and “how to face a challenging clinical situation”. Post-qualification training should nurture the trainee into the art and science of mentorship so that they can impart effective education to the future generations of orthopedic trainees. The evaluation of the trainees during their qualification should be objective and structured to the course content and not unlimited as the current practice.

We are in the era of information technology, where a plethora of information is available for use. Some times excessive information also creates confusion and doubts in the mind of the user. It is important to draw reliable conclusions for everyday clinical practice. The conclusions based on scientific analysis of the literature stand the test of time and predictably help to plan management. The present issue has review article by Handoll et al. on “The Cochrane Collaboration: a leading role in producing reliable evidence to inform healthcare decisions in musculoskeletal trauma and disorders” highlighting the methodology of the Cochrane collaboration in generating evidence for clinical practice. Systematic reviews of articles and Randomized controlled trails(RCTs) are important to develop evidence-based guidelines. The very bases of such reviews are defining clear research questions, analysis of eligible studies collected by an explicit, and predefined systematic search of the literature and analyzing scientifically to draw robust evidence.

This implies that for systematic reviews, sufficient articles/RCTs for a specific research question are required. The publication of studies depends on the prevailing clinical problems in a geographical area, desire of clinicians to analyze the clinical data, infrastructure to conduct the studies and publication in retrievable (indexed) journals.

The clinical problems of developing countries are diagonally different from those of developed nations.^8^ While the developed countries mainly treat congenital, traumatic and degenerative pathologies, developing countries still face infections, traumatic, nutritional and late presentation of various musculoskeletal disorders and sequalae of inadequately, improperly treated musculoskeletal problems. This difference is reflected in the published literature clearly. During the last five years, 4604 articles were retrieved in the English literature in “PubMed” on total hip arthroplasty while only 240 on acute osteomyelitis, 339 on spinal tuberculosis, 354 articles were published on clubfoot while eight on neglected clubfoot. The articles published by authors from India and other developing countries are very few. This difference is also reflected in this issue of Indian Journal of Orthopaedics. Out of seven articles on total hip replacement, four from developed countries are covering minimally invasive total hip replacement, bone ingrowth on a tantalum cup and review article on unstable total hip replacement, while three studies from India report salvage from failed fixation of proximal femur fractures or proximally deficient femur or a conversion of failed hemiarthroplasty. The evidence for the management of fresh femoral neck fracture may be sound and available but for neglected femoral neck fracture are still to be created particularly when neglect could be of three weeks, three months, six months or more in young patients. We need to clearly define the objective and expectation from treatment in different subgroups with guidelines particularly keeping in mind the geographical social milieu. The preservation of the femoral head with some limitation may still be a better option than total hip arthroplasty particularly for a population having an infrastructure and resource crunch and where even social milieu may not go well with replacement arthroplasty.

The surgeons in developing countries are burdened by a large number of patients, poor infrastructure to treat and conduct research, less incentives for research and lack of enthusiasm to publish. Most of the indexed journals are published from developed countries and are less inclined to accept articles on the problems of developing countries. With the present approach of the “Cochrane group” it looks an improbable task to generate the evidence when published data is not available from developing countries on their clinical problems.

This can be done by creating a collaborative of orthopedic surgeons who are desirous to create evidence. The collaborative should include “subspecialties” of the musculoskeletal system. The list of research questions, the research strategies to find out available evidence needs to be drawn, failing which specific research is to be conducted to generate evidence for day-to-day clinical problems. This will help in formulating guidelines for the management of various musculoskeletal disorders in developing countries.

The developing countries will have to work in multiple directions. At one end they have to work hard to generate evidence for improving patient care in their available infrastructure at other end they need to keep pace with west in upgrading orthopaedic education, so that the future surgeons are prepared to take the lead role in research, generating evidence to improve patient care at large.
